# Use of Intravenous Peramivir for Treatment of Severe Influenza A(H1N1)pdm09

**DOI:** 10.1371/journal.pone.0040261

**Published:** 2012-06-29

**Authors:** Janice K. Louie, Samuel Yang, Cynthia Yen, Meileen Acosta, Robert Schechter, Timothy M. Uyeki

**Affiliations:** 1 California Department of Public Health, Richmond, California, United States of America; 2 Influenza Division, Centers for Disease Control and Prevention, Atlanta, Georgia, United States of America; University of Calgary & ProvLab Alberta, Canada

## Abstract

Oral antiviral agents to treat influenza are challenging to administer in the intensive care unit (ICU). We describe 57 critically ill patients treated with the investigational intravenous neuraminidase inhibitor drug peramivir for influenza A (H1N1)pdm09 [pH1N1]. Most received late peramivir treatment following clinical deterioration in the ICU on enterically-administered oseltamivir therapy. The median age was 40 years (range 5 months-81 years). Common clinical complications included pneumonia or acute respiratory distress syndrome requiring mechanical ventilation (54; 95%), sepsis requiring vasopressor support (34/53; 64%), acute renal failure requiring hemodialysis (19/53; 36%) and secondary bacterial infection (14; 25%). Over half (29; 51%) died. When comparing the 57 peramivir-treated cases with 1627 critically ill cases who did not receive peramivir, peramivir recipients were more likely to be diagnosed with pneumonia/acute respiratory distress syndrome (p = 0.0002) or sepsis (p = <0.0001), require mechanical ventilation (p = <0.0001) or die (p = <0.0001). The high mortality could be due to the pre-existing clinical severity of cases prior to request for peramivir, but also raises questions about peramivir safety and effectiveness in hospitalized and critically ill patients. The use of peramivir merits further study in randomized controlled trials, or by use of methods such as propensity scoring and matching, to assess clinical effectiveness and safety.

## Introduction

The influenza A(H1N1)pdm09 [pH1N1] virus emerged in April 2009 to cause a global pandemic [1]–[3]. In California, the reported morbidity and mortality were high, with 2042 persons admitted to intensive care units (ICU); 609 were fatal.

Early in the pandemic, the only available antiviral drugs for treatment of pH1N1 were the neuraminidase inhibitors (NAI), oral oseltamivir or inhaled zanamivir. Administration of enterically- administered oseltamivir can be challenging in the ICU where patients may have difficulty tolerating oral administration of capsules or intestinal ileus may cause malabsorption [4]. Use of inhaled zanamivir is not formulated for use in patients on mechanical ventilation [2]. As part of the emergency public health response, on November 19, 2009 the Commissioner of the Food and Drug Administration issued an Emergency Use Authorization (EUA) for the experimental intravenous (IV) NAI drug peramivir. Under the EUA, peramivir was distributed by the Centers for Disease Control and Prevention (CDC) for treatment of hospitalized pediatric and adult patients with evidence of pH1N1 virus infection and who 1) were not responding to antiviral therapy, or 2) the clinician deemed that enteral or inhaled delivery of NAIs was not dependable or feasible [5].

In April 2009, the California Department of Public Health (CDPH) initiated mandatory reporting for critically ill and fatal cases of pH1N1. In this report, we describe the epidemiology, clinical characteristics, and outcomes of pH1N1 cases reported to CDPH who received IV peramivir during the pandemic.

## Methods

This study protocol was reviewed and approved by the State of California Committee for the Protection of Human Subjects (CPHS). A waiver of informed consent was granted as the data were collected as part of public health practice and were analyzed anonymously, the risk to subjects was minimal, the rights and welfare of subjects were not adversely affected, and the research could not have been practically carried out without a waiver (Common Rule 45 CFR 46.11).

A case was defined as a California resident who was hospitalized in an ICU for ≥24 hours with signs and symptoms consistent with acute respiratory infection and laboratory evidence of pH1N1 virus infection by reverse-transcription polymerase chain reaction (RT-PCR). Cases were reported by providers, hospitals and county coroners to local health jurisdictions (LHJ) who subsequently reported them to CDPH. Data on demographics, clinical presentation and hospital course, co-morbid conditions, dosing and dates of antiviral medications administered were abstracted from medical records, pharmacy medication records and autopsy reports by staff at the LHJ or CDPH using the same standardized case report form. Secondary bacterial co-infection was defined by isolation of bacteria within 3 days of admission from either a sterile site (e.g., blood, cerebrospinal fluid, or pleural fluid) or a lower respiratory tract specimen (e.g., bronchoalveolar lavage or endotracheal aspirate) in conjunction with new infiltrate on chest radiograph.

Comparative analysis was performed for 1) critically ill patients who received peramivir and critically ill patients who were treated with NAIs but did not receive peramivir; and 2) fatal and non-fatal peramivir-treated cases, with respect to demographics, clinical characteristics, and underlying risk factors; differences in categorical variables were analyzed using the chi-square test for comparisons of categorical variables with large numbers and the Fisher’s exact test for comparisons of categorical variables with numbers <5. All analyses were performed using SAS 9.2 (SAS Institute, Cary, NC).

## Results

During April 3, 2009 – August 10, 2010, 1962 California residents were reported with pH1N1 virus infection that were hospitalized ≥24 hours and required intensive care. Information on whether or not antiviral treatment was given was available in 1867 (95%) cases; of these, 1684 (90%) received treatment with an NAI.

Fifty-seven (3%) cases received IV peramivir (Table 1). Fifty-two of these cases received oseltamivir as a second antiviral, and another five cases were treated with peramivir plus two or more other antivirals [e.g. oseltamivir and amantadine (1), oseltamivir and inhaled zanamivir (1), oseltamivir and rimantadine (2), and amantadine and ribavirin (1)]. Fifty-one cases had information available on timing of oseltamivir and peramivir treatment: 14 (28%) received oseltamivir prior to initiation of peramivir, and 37 (73%) received concurrent administration of oseltamivir and peramivir for least 24 hours; the median time for concurrent administration of both drugs was one day (range 1–14); 19 (51%) of these 37 died. The median number of days from illness onset to starting treatment with an oral NAI or IV peramivir were 4 days (range 0–23) and 9 days (range 2–38), respectively. The most common reasons cited in the medical records for initiation of peramivir included “not responding to oral or inhaled antivirals” (43; 75%) followed by “suspected malabsorption” (7; 12%).

**Table 1:** Critically ill and fatal cases with 2009 pandemic influenza A (H1N1) treated with intravenous peramivir in California, April 2009–August 2010.

Variable	Received peramivir 1 (n = 57)	Fatal (n = 29)	Non-fatal (n = 28)	*P*-value 2
**Demographics**				
Female sex	26 (46%)	12 (41%)	41.5 (0.42–77)	NS
Median age (years) and range	40 (0.42–81)*	41 (10–81)	41.5 (0.42–77)	NS
Race/ethnicity^3^				NS
Hispanic	22 (39%)	12 (41%)	10 (36%)	
White, Non-Hispanic	22 (39%)	9 (31%)	13 (46%)	
Asian/Pacific Islander	4 (7%)	2 (7%)	2 (7%)	
Black, Non-Hispanic	7 (12%)	5 (17%)	2 (7%)	
Other	2 (4%)	1 (3%)	1 (4%)	
Native American	0 (0%)	0 (0%)	0 (0%)	
Unknown	0	0	0	
**ACIP Co-morbid conditions** 3,4				
Any ACIP comorbidity	41 (72%)	20 (69%)	21 (75%)	NS
Chronic cardiac disease^5^	15 (26%)	8 (28%)	7 (25%)	NS
Chronic lung disease^6^	30 (53%)	16 (55%)	14 (50%)	NS
Asthma	13 (23%)	7 (24%)	6 (21%)	NS
Metabolic disease^7^	18 (32%)	11 (38%)	7 (25%)	NS
Diabetes mellitus	12 (21%)	7 (24%)	5 (18%)	NS
Renal Disease	7 (12%)	3 (10%)	4 (14%)	NS
Neurologic disorder^8^	3 (5%)	0 (0%)	3 (11%)	NS
Immunosuppressive conditions^9^	12 (21%)	7 (24%)	5 (18%)	NS
Pregnant	3/26 (12%)	2/12 (17%)	1/14 (7%)	NS
Obesity^10^	26/46 (57%)	15/24 (63%)	11/22 (50%)	NS
BMI ≥40	12/26 (46%)	8/15 (53%)	4/11 (36%)	NS
**Clinical complications and management**				
Pneumonia/ARDS	55 (96%)	29 (100%)	26 (93%)	NS
Mechanical ventilation	54 (95%)	27 (93%)	27 (96%)	NS
Sepsis requiring vasopressors^1^	34/53 (64%)	21/28 (75%)	13/25 (52%)	NS
Acute renal failure requiring hemodialysis	19/53 (36%)	14/28 (50%)	5/25 (20%)	0.023
ECMO	9/55 (16%)	5/28 (18%)	4/27 (15%)	NS
Pulmonary embolus	1 (2%)	1 (3%)	0 (0%)	NS
Secondary bacterial infection^11^	14 (25%)	7 (24%)	7 (25%)	NS
Death	29 (51%)	29 (100%)	N/A	N/A
Length of hospital stay in days, median (range)	21 (3–78)	16 (3–68)	31 (11–78)	0.002
Length of ICU stay in days, median (range)	12.5 (1–66)	12 (1–66)	13 (13–13)	NS
Time from hospital admission to ICU in days, median (range)	0 (0–5)	0 (0–3)	0 (0–5)	NS
**Details of medical treatment, including antivirals**				
Steroids^1^	36/52 (69%)	21/28 (75%)	15/24 (63%)	NS
Intravenous Immunoglobulin (IVIG)^1^	9/51 (18%)	4/27 (15%)	5/24 (21%)	NS
Treated with peramivir and oseltamivir 12	56 (98%)	28 (97%)	28 (100%)	NS
Treated with peramivir and double-dose oseltamivir^13^	23/56 (41%)	8/28 (29%)	15 (54%)	NS
Treated with peramivir plus two or more other antivirals^14^	5 (9%)	4 (14%)	1 (4%)	NS
Time from symptom onset to any antiviral treatment, in days, median (range)^3^	4 (0–23)	5 (0–23)	4 (0–9)	NS
Time on any antiviral treatment, in days, median (range)^3^	13 (3–36)	12 (3–36)	13.5 (9–33)	NS
Time from symptom onset to peramivir treatment, in days, median (range)^3^	9 (2–38)	10 (2–36)	9 (2–38)	NS
Time on peramivir treatment, in days, median (range)^3^	8 (1–22)	7 (1–15)	9 (5–22)	0.0225

**NOTE.** Data are no. (%) of case patients, unless otherwise indicated. ACIP: Advisory Committee on Immunization Practices, BMI: body mass index, ARDS: Acute respiratory distress syndrome, N/A: Not Applicable, ICU: intensive care unit, ECMO: Extracorporeal membrane oxygenation.

1 Defined as cases who received at least one dose.

2 Comparing fatal and non-fatal peramivir recipients.

3 Includes case patients with known information only.

4 Conditions listed are not mutually exclusive because of the presence in some patients of multiple underlying chronic diseases.

5 Includes coronary arterial disease, congestive heart failure, congenital heart disease, arrythmia, and other/unknown cardiac disease.

6 Includes asthma, obstructive sleep apnea, chronic obstructive pulmonary disease, or other chronic pulmonary disease.

7 Includes diabetes, renal disease, chronic renal insufficiency, end-stage renal disease, hemodialysis, continuous ambulatory peritoneal dialysis, hypothyroidism, or other metabolic diseases.

8 Includes seizure disorder, cerebral palsy/developmental delay, or other neurological disorders.

9 Includes immunosuppressive drugs, cancer, chemotherapy, leukemia, systemic lupus erythematosus, transplant, or other immunosuppressive conditions.

10 Obesity only counted if body mass index data are available and age ≥20 years.

11 Includes *Staphylococcus aureus* of all susceptibility patterns, group A *Streptococcus, Streptococcus pneumoniae*, gram-negative rods, and other bacteria.

12 Defined as a case who received at least one dose of peramivir and at least one dose of oseltamivir (75 mg).

13 Defined as a case who received at least one dose of peramivir and one double dose of oseltamivir (150 mg).

14 Five cases were treated with peramivir plus two or more other antiviral drugs including amantadine and ribavirin (1), oseltamivir and amantadine (1), oseltamivir and zanamivir inhaled (1), and oseltamivir and rimantadine (2).

* Ten peramivir recipients were <18 years. Seven (70%) had underlying medical conditions including chronic pulmonary (4), cardiac (4) and neurologic (3) disorders. Complications included pneumonia/ARDS (9;90%), sepsis (6;60%) and secondary bacterial co-infection (4;40%). Supportive measures given included mechanical ventilation (10;100%), hemodialysis (3/9;33%) and ECMO (4;40%). Two (20%) children died.

The median age of peramivir recipients was 40 years (range 5 months-81 years). Fifty-six of 57 (98%) received antibiotic treatment at admission to the hospital. Almost all (55; 96%) had radiographic evidence of pneumonia with 54 (95%) requiring mechanical ventilation. Fifty-three cases had information on dates of antiviral treatment: 34 (64%) required vasopressor support for hypotension associated with sepsis and 19 (36%) had new onset acute renal failure requiring hemodialysis. Fourteen (25%) had microbiologic evidence of community-acquired bacterial co-infection at admission. The median length of hospital stay was 21 days (range 3–78).

Twenty-nine (51%) peramivir recipients died. Fatal peramivir recipients differed from non-fatal peramivir recipients by being more likely to be diagnosed with acute renal failure requiring hemodialysis (p = 0.02), to have a shorter length of hospital stay (median 16 days vs. 31 days; p = 0.002) and to receive peramivir for a shorter duration (7 days vs. 9 days; p = 0.02). There was no significant difference between time from onset of symptoms and initiation of any antiviral treatment among fatal and non-fatal peramivir recipients (Table 1).

We also compared the 57 peramivir recipients to the 1627 cases in the ICU who were treated with other NAIs but did not receive peramivir. There was no significant difference with regard to the overall proportion with an ACIP co-morbid condition, although cases treated with peramivir had a slightly higher prevalence of obesity (p = 0.04) and morbid obesity (p = 0.02). Peramivir recipients were more likely to be diagnosed with pneumonia/ARDS (p = 0.0002) or sepsis (p = <0.0001), to require mechanical ventilation (p = <0.0001) and to die (p = <0.0001). Both peramivir and non-peramivir recipients were initiated on NAIs within similar timeframes [median 4 days (range 0–23) and 4 days (range 0–52), respectively; p = 0.6].

## Discussion

Our report adds to the limited existing data on use of the investigational drug peramivir in hospitalized and critically ill influenza patients. Two small case-series have described use of multi-dose peramivir during the pH1N1 pandemic; one study of 31 critically ill adults and children conducted prior to the EUA found a 56-day mortality of 41% (59% survival) [6], while another report of 41 cases identified from multi-site hospitalization data reported 27% mortality [7]. Clinical trials in hospitalized seasonal influenza patients found no significant difference in treatment outcome between five day courses of peramivir and either placebo or oseltamivir [8]. The same has been found in the outpatient setting; in clinical trials of over 1000 healthy adults treated with either a single infusion of peramivir or a five day course of oral oseltamivir, no difference was found in duration of symptoms (peramivir recipients had decreased viral shedding) [9]. In our series, over half of peramivir-treated cases died despite prolonged antiviral treatment and aggressive supportive measures. Critically ill cases who received peramivir were more likely to die than those who did not receive peramivir.

The case fatality proportion (51%) in our pH1N1 patient population was higher than that reported in other peramivir case series, but comparisons are impeded by the limited clinical data available. Of note, the 57 patients in our study were older (median age of 40 vs. 23 years) and had a higher prevalence of co-morbidities (72% vs. 45%) than the 31 patients who received peramivir prior to the EUA (overall 41% mortality) [6]. Likewise, in a second study of 41 peramivir patients with 27% case fatality [7], the patients in our study had a higher proportion of ICU admission (100% vs. 93%), mechanical ventilation (95% vs. 90%), chronic pulmonary disease (53% vs. 29%), diabetes (21% vs. 12%), obesity (57% vs. 44%), and morbid obesity (46% vs. 17%) [7]. More data are needed to fully understand the impact of these demographic and clinical differences on overall mortality.

The high case fatality proportion described in our patient population may have been due to the progressive severity of illness; the majority of requests for peramivir were for cases assessed by the clinician as not responding to several days of oral oseltamivir therapy and with signs of continued clinical deterioration. However, it also raises concerns of adverse drug events specifically due to peramivir. Most of the available safety data describes single-dose use of peramivir in adult and pediatric outpatient populations; commonly reported adverse events have included diarrhea, nausea, vomiting, and neutropenia [5], [8], [10]. Limited data are available on tolerability of multiple-dose peramivir in critically ill patients. Reassuringly, while the nature of our surveillance does not allow for detailed review, none of the peramivir recipients in the medical records we reviewed had adverse events, side effects or fatality that was assessed by the clinician as being a result of peramivir administration, including nine children and three pregnant women. This is consistent with limited descriptive reports from other institutions during the pandemic [6]–[7].

Additionally, in our series, 37 critically ill cases received concurrent administration of oseltamivir and peramivir for more than 24 hours, with over half dying. The pH1N1 pandemic highlighted concerns that widespread use of a single antiviral agent could lead to emergence of resistant strains. Treatment with a combination of two antiviral agents could theoretically offer the advantages of additive effect, while decreasing severity of disease, duration of viral shedding and risk of developing resistance. However, because all drugs in the NAI class target the same binding pocket, drug antagonism is also a concern. Recent reports suggest that combination NAI therapy may be less effective than monotherapy; in a randomized controlled trial of over 500 outpatients with pH1N1 influenza, treatment with concurrent oral oseltamivir and inhaled zanamavir was associated with more side effects (nausea and vomiting) and shedding of higher viral titers for longer periods compared to those receiving either drug alone [11]. Another case-series describing 21 critically ill patients treated with oseltamivir and IV zanamavir reported a 24% mortality and prolonged viral shedding [12]. Until more data are available, combination NAI therapy should be used with caution.

Some limitations are important to note. There was likely underreporting of cases during the pH1N1 pandemic, including those who received peramivir. While 57 cases were identified by search of the CDPH surveillance database, during the same timeframe the CDC received 130 requests for peramivir from California [CDC, unpublished data.], suggesting that treatment was not administered to many cases, due to clinical improvement, death, or other reasons. Serial testing for influenza by PCR, which might inform decisions on when to modify or cease antiviral therapy, was not performed in our 57 peramivir recipients. It is possible that some patients may have had poor outcomes following peramivir treatment related to the presence of the H275Y mutation in viral neuraminidase, which confers resistance to oseltamivir and reduced in-vitro susceptibility to peramivir [13]. This is likely a rare event; during the period of this surveillance, of 2260 cases tested, only nine cases of infection with pH1N1 viruses containing the H275Y mutation were identified in California (CDPH, unpublished data).

In conclusion, current guidelines strongly recommend treatment with oral oseltamivir as soon as possible in influenza patients who are hospitalized, have ACIP-defined high risk conditions, or have severe or progressive clinical presentation [14]–[15]. For critically ill patients in the ICU, investigational IV NAIs such as peramivir or IV zanamivir may be an option; both are available through clinical trials (http://clinicaltrials.gov) and IV zanamivir is also available on a compassionate use basis (http://www.clinicalsupporthd.gsk.com/). However, while the high mortality (>50%) we observed could be due to the pre-existing clinical severity of cases prior to request for peramivir, it also raises questions about peramivir safety and effectiveness in hospitalized and critically ill patients. Although ours is the largest case-series describing the use of peramivir in criticaly ill patients published to date, the nature of our surveillance and the criteria for requesting peramivir under EUA precludes any comparison of effectiveness between the oral and IV NAIs. Large randomized clinical trials are required to assess these questions appropriately among hospitalized influenza patients, or, in the absence of data from randomized controlled trials by use of methods such as propensity scoring and matching to assess the clinical effectiveness and safety of new investigational agents for influenza treatment.
